# Higher Food Inflammation Index Is Linearly Associated With Higher Risk of MASLD: A Cross‐Sectional Study Based on the NHANES (1999–2020)

**DOI:** 10.1002/fsn3.70865

**Published:** 2025-09-02

**Authors:** Qingwan Yang, Xin Cai, Zhenghua Xiao

**Affiliations:** ^1^ The Second Clinical Medical College of Guizhou University of Traditional Chinese Medicine Guiyang China; ^2^ Department of Rheumatology and Immunology Guiyang First People's Hospital Guiyang China; ^3^ Department of Gastroenterology The Second Affiliated Hospital of Guizhou University of Traditional Chinese Medicine Guiyang China

**Keywords:** Dietary Inflammatory Index (DII), dose–response relationship, Food Inflammatory Index (FII), metabolic dysfunction‐associated steatotic liver disease (MASLD), National Health and Nutrition Examination Survey (NHANES)

## Abstract

Metabolic dysfunction‐associated steatotic liver disease (MASLD) is a major global chronic liver condition, with diet‐induced inflammation playing a key role in its pathogenesis. This study used 1999–2020 National Health and Nutrition Examination Survey (NHANES) data to assess the association between the Food Inflammation Index (FII) and MASLD risk compared with the Dietary Inflammatory Index (DII). The FII, which is based on 39 food components (via two 24‐h dietary recalls), is simpler than the DII (45 components). MASLD was defined as a Fatty Liver Index (FLI ≥ 60) with at least one cardiometabolic risk factor. This study included 25,067 participants (mean age: 49.56 years; 51.51% Male), of whom 6708 met the MASLD diagnostic criteria. Multivariable logistic regression revealed that a 1‐standard deviation (SD) increase in the FII was associated with a 7.9% higher MASLD risk (OR = 1.079, 95% CI: 1.025–1.137; *p* = 0.004) in the fully adjusted model. Compared with the lowest quartile (Q1), the highest FII quartile showed a 30.8% increased risk (OR = 1.308, 95% CI: 1.143–1.496; *p* < 0.001). Restricted cubic splines confirmed a linear dose–response relationship (*p*‐overall < 0.001; P‐nonlinearity = 0.545). Subgroup analyses confirmed consistent FII‐MASLD associations across diverse populations (*p*‐interaction > 0.05), with five sensitivity analyses verifying robustness. The area under the curve (AUC = 0.867) of the FII was comparable to that of the DII (AUC = 0.866), but the FII improved risk reclassification by 5.56% (Net Reclassification Improvement [NRI] = 0.0556; *p* = 0.002) and discrimination by 0.05% (integrated discrimination improvement [IDI] = 0.0005; *p* = 0.015). The FII effectively assesses dietary inflammation in patients with MASLD, supporting targeted nutritional interventions, but further validation is needed.

## Graphical abstract

A higher Food Inflammation Index (FII) is associated with an increased risk of metabolic dysfunction‐associated steatotic liver disease (MASLD) (OR = 1.079 per 1‐SD increase, *p* = 0.004). The FII, based on 39 components, has comparable predictive accuracy to the DII (AUC = 0.867 vs. 0.866) but superior risk reclassification (NRI = 0.0556, *p* = 0.002). The FII effectively detects dietary inflammation, enabling targeted nutritional interventions for MASLD.

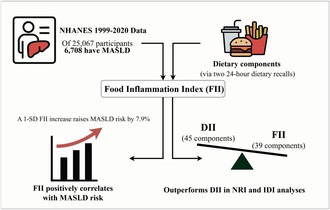

**Abbreviations**
AUC: area under the curve
BMI: body mass index
CI: confidence interval
CRP: C‐reactive protein
DII: dietary inflammatory index
FII: food inflammation index
FLI: fatty liver index
FNDDS: Food and Nutrient Database for Dietary Studies
GGT: gamma‐glutamyl transferase
HDL‐C: high‐density lipoprotein cholesterol
IDI: integrated discrimination improvement
MASLD: metabolic dysfunction‐associated steatotic liver disease
MAFLD: metabolic dysfunction‐associated fatty liver disease
MET: metabolic equivalent of task
NAFLD: nonalcoholic fatty liver disease
NHANES: National Health and Nutrition Examination Survey
NRI: net reclassification improvement
NRV: nutritional recommended value
OR: odds ratio
PA: physical activity
PIR: poverty‐to‐income ratio
RCS: restricted cubic spline
ROC: receiver operating characteristic
SD: standard deviation
TG: triglycerides
TIS: total inflammation score
TNF: tumor necrosis factor
WC: waist circumference

## 1 Introduction

Metabolic dysfunction‐associated steatotic liver disease (MASLD), formerly referred to as nonalcoholic fatty liver disease (NAFLD) and metabolic dysfunction‐associated fatty liver disease (MAFLD), is a global public health challenge affecting more than one‐third of adults worldwide (Rinella et al. 2023; Miao et al. 2024). MASLD is characterized by excessive lipid accumulation in hepatocytes, excluding significant alcohol consumption, viral infections, drug toxicity, or autoimmune liver injuries. It encompasses a spectrum from simple steatosis to steatohepatitis, fibrosis, cirrhosis, and hepatocellular carcinoma (Hagström et al. 2024; Israelsen et al. 2024). In 2019, MASLD affected nearly 1 billion people globally, reflecting a 111.79% increase in prevalence since 1990 (Lv et al. 2024). Regional variations, with higher prevalence in Asian populations, underscore the need for context‐specific interventions (Younossi et al. 2025). As the primary cause of chronic liver disease and a growing contributor to liver‐related mortality, MASLD imposes a substantial socioeconomic burden. Despite its increasing prevalence, no pharmacological treatments exist, making lifestyle interventions—particularly dietary optimization—central to MASLD prevention and management (Semmler et al. 2023; Armandi and Bugianesi 2024).

MASLD is increasingly recognized as a chronic inflammatory condition driven by metabolic dysfunction (Byrne et al. 2025). Diet, a key modifiable risk factor, influences MASLD progression through its pro‐ or anti‐inflammatory effects (Liu et al. 2025). Unlike Mediterranean diets, which mitigate inflammation, Western diets exacerbate it (Adolph and Tilg 2024; Liu et al. 2024). Chronic low‐grade inflammation induced by dietary components is linked to hepatic steatosis and fibrosis, providing tools for quantifying dietary inflammatory effects (Sedighi et al. 2025).

Introduced in 2014, the Dietary Inflammatory Index (DII) is a validated tool that assesses dietary inflammatory potential based on 36 anti‐inflammatory and nine proinflammatory food components (Shivappa et al. 2014). Cohort studies and a cross‐sectional analysis of 2383 US adults with MASLD have linked higher DII scores to increased MASLD risk (Xu et al. 2024). In children aged 7–18 years, the DII is significantly positively correlated with the MASLD score. Each unit increase in the DII score is associated with a 2.6‐fold higher likelihood of severe hepatic steatosis (Amiri et al. 2025). Refinements such as the energy‐adjusted E‐DII (Hébert et al. 2019) and adolescent‐specific C‐DII (Khan et al. 2018) have enhanced its global and demographic applicability. However, the DII's reliance on nutrient intake data without standardized portion‐size adjustments and lack of consideration for food group heterogeneity restricts its precision in capturing dietary inflammation (Hébert et al. 2019). Notably, the DII's limited focus on various types of monounsaturated fatty acids (MUFAs) and omega‐3 polyunsaturated fatty acids (PUFAs), among other bioactive fatty acids, restricts its sensitivity in capturing dietary inflammation in metabolic diseases, including MASLD and type 2 diabetes, where omega‐3 PUFAs are crucial (Hébert et al. 2019; Gorczyca et al. 2024; Wang et al. 2025).

To address these limitations, the Food Inflammation Index (FII) was developed in 2024 as a refined tool to evaluate dietary inflammatory potential (Wang et al. 2025). Compared with the DII, the FII offers several advancements: (1) a standardized scoring method using percentage calculations for consistent assessment of foods and beverages; (2) the inclusion of multiple anti‐inflammatory food components, such as PUFAs and MUFAs, for detailed analysis of inflammatory effects; (3) the consideration of intra‐food group variability (e.g., distinguishing anti‐inflammatory nuts from proinflammatory processed meats); and (4) a flexible scoring approach adaptable to diverse dietary patterns, validated with 29 Mediterranean diet components (Wang et al. 2025). These features enable more precise and nuanced dietary recommendations. The DII's association with MASLD is established, but the FII's relationship with MASLD remains unexplored, representing a critical knowledge gap. Compared with the DII, the FII, a refined tool, offers greater precision in analyzing intra‐food group variability and anti‐inflammatory components, such as omega‐3 fatty acids. Its evaluation in MASLD could refine dietary strategies for prevention and management and provide novel insights into dietary inflammation indices in metabolic diseases.

This study investigates the association between FII scores and MASLD risk among National Health and Nutrition Examination Survey (NHANES) participants, using the Fatty Liver Index (FLI) as the diagnostic criterion. We hypothesize that higher FII scores, indicating greater dietary inflammatory potential, are positively associated with increased MASLD risk. By clarifying this relationship, our findings could enhance dietary assessment strategies for MASLD prevention and management, providing a novel tool for clinical and public health interventions.

## 2 Methods

### 2.1 Study Design

**FIGURE 1:**
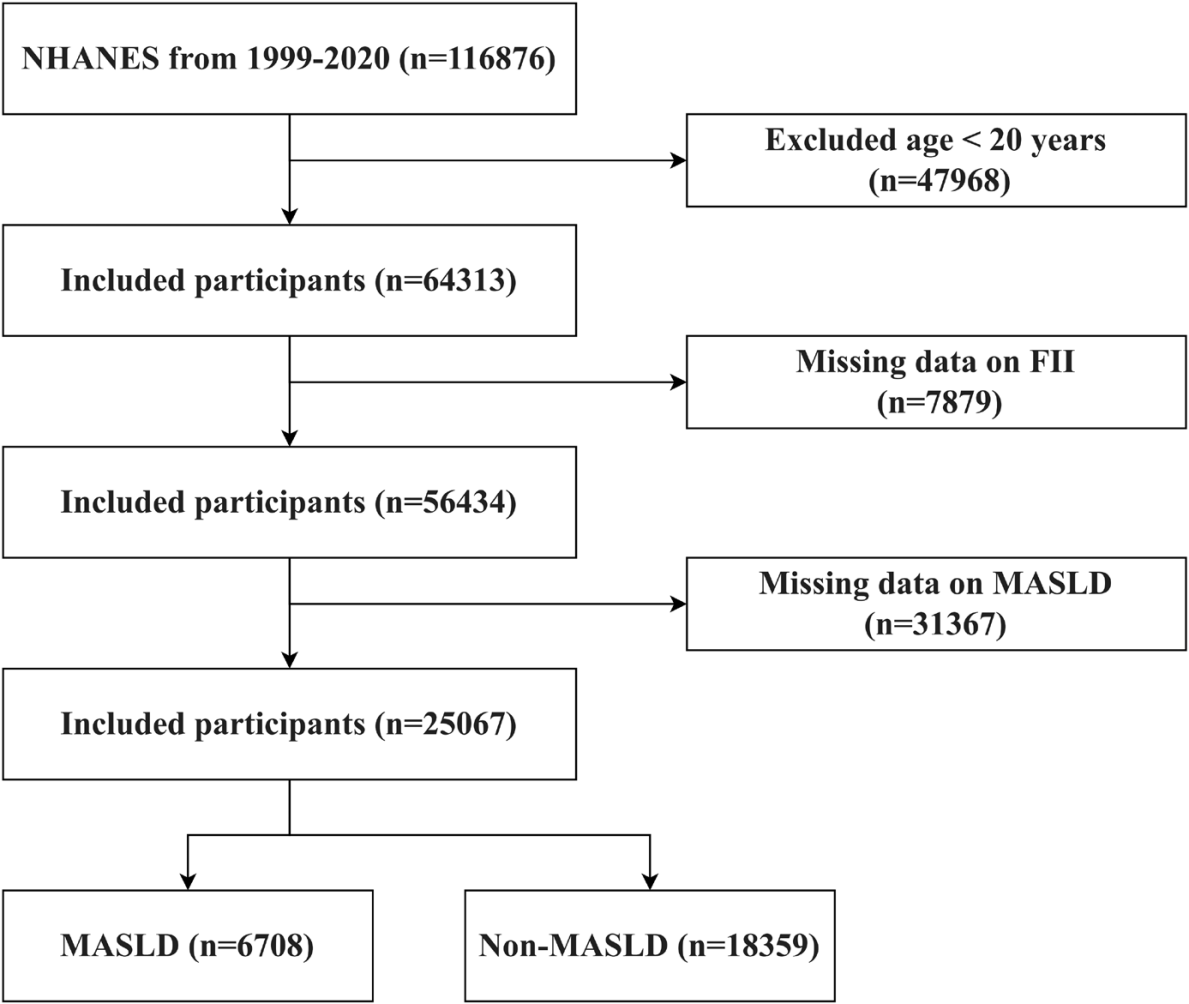
Flowchart depicting the selection of participants.

We extracted data from the NHANES database, a nationally representative cross‐sectional survey assessing the health and nutritional status of the U.S. population, using a complex, multistage probability sampling design (Zipf et al. 2013). In accordance with the National Center for Health Statistics Ethics Review Board, all participants provided written informed consent, eliminating the need for additional ethical approval. This study utilized data from NHANES surveys conducted between 1999 and 2020, which included participants aged 20 years or older. The initial sample comprised 116,876 participants. We excluded those under 20 years (*n* = 52,563), those with incomplete FII data (*n* = 7879), and those with missing MASLD data (*n* = 31,367). This yielded a final cohort of 25,067 individuals, including 6708 with MASLD (Figure 1).

### 2.2 Assessment of Dietary Intake

Dietary intake data were derived from NHANES dietary recall data. Nutrient intake was assessed using two nonconsecutive 24‐h dietary recalls: the first conducted in person and the second via telephone 3–10 days later (Ahluwalia et al. 2016). Dietary data were analyzed using the USDA Food and Nutrient Database for Dietary Studies (FNDDS) to estimate daily nutrient intake, with the average calculated from the two‐day recalls.

### 2.3 Assessment of the DII

The DII, which is based on 45 dietary components (Table S1), uses six inflammatory markers (IL‐1β, IL‐6, CRP, TNF‐α, IL‐4, and IL‐10) to score inflammatory effects. Components increasing the levels of proinflammatory markers (IL‐1β, IL‐6, CRP, and TNF‐α) or decreasing the levels of anti‐inflammatory markers (IL‐4 and IL‐10) score +1 or −1, respectively; neutral components score 0. Positive DII scores indicate proinflammatory potential (Shivappa et al. 2014). The DII is calculated by converting intakes to *Z* scores and then percentiles, multiplying by inflammatory effect scores, and summing all component scores (Shivappa et al. 2014).

### 2.4 Assessment of the FII

The FII, an enhanced version of the DII, aims to quantify dietary inflammatory potential with greater precision, addressing food group heterogeneity. The FII incorporates 39 dietary components, excluding culinary ingredients (e.g., rosemary, onions, and pepper), to focus on primary food sources, with omega‐3 PUFAs (α‐linolenic acid, eicosapentaenoic acid, and docosahexaenoic acid), as detailed in Table S2 (Wang et al. 2025). FII scores were calculated from 24‐h dietary recalls with portion‐size standardization to reduce recall bias, which is a limitation of the DII (Shivappa et al. 2014). Intakes were converted to *z* scores relative to NHANES medians, weighted by Total Inflammation Scores (TISs) and Nutritional Recommended Values (NRVs) from the 2020–2025 Dietary Guidelines for Americans (ages 31–50 years) and global intake data.

FII scores were calculated in two steps: First, the FII was derived from food components, TISs, and NRVs using Equation (1):

(1) $$\mathrm{FII}/100\;g=\sum_{i=1}^{n}\frac{\text{TISi}\times \mathrm{Ni}}{\text{NRVi}}$$

where *TISi* denotes the total inflammatory score for each dietary component (Semmler et al. 2023) and *Ni* represents the content per 100 g of food for that component. *NRVi* was based on the recommended dietary allowances for adults aged 31–50 years from the 2020–2025 Dietary Guidelines for Americans, supplemented by global intake data for uncovered nutrients (Table S2).

To minimize outlier effects, food items were truncated to the 1st–99th percentile range. The normalized FII score was calculated using Equation (2):

(2) $$\mathrm{FII}\;\text{score}=100-\mathrm{abs}\left(\frac{\mathrm{FII}-\mathrm{FII}\left(99\%\right)}{\mathrm{FII}\left(1\%\right)-\mathrm{FII}\left(99\%\right)}\right)\times 99$$

Here, FII (99%) and FII (1%) represent the values at the 99th and 1st percentiles of the FII distribution, respectively.

### 2.5 Definition of MASLD

Hepatic steatosis was defined using the FLI, with MASLD diagnosed at an FLI score ≥ 60. The FLI, developed by Bedogni et al. (Bedogni et al. 2006), is a noninvasive tool that relies on clinical and laboratory parameters such as body mass index (BMI), waist circumference (WC), triglycerides (TGs), and gamma‐glutamyl transferase (GGT). The FLI was calculated using Equation (3):

(3) $$\mathrm{FLI}=\frac{e\left(0.953\cdot \ln\left(\mathrm{TG}\right)+0.139\cdot \mathrm{BMI}+0.718\cdot \ln\left(\mathrm{GGT}\right)+0.053\cdot \mathrm{WC}-15.745\right)}{\left(1+e\left(0.953\cdot \ln\left(\mathrm{TG}\right)+0.139\cdot \mathrm{BMI}+0.718\cdot \ln\left(\mathrm{GGT}\right)+0.053\cdot \mathrm{WC}-15.745\right)\right)}\times 100$$

where ln is the natural logarithm, e is its base, *TG* is in mmol/L, *GGT* is in U/L, and WC is in cm. The resulting score ranges from 0 to 100.

Compared with invasive methods such as liver biopsy, the FLI is safer, simpler, and more cost‐effective, making it ideal for large‐scale population screening and epidemiological studies. Multiple studies have validated its high sensitivity and specificity for diagnosing fatty liver (Bedogni et al. 2006; Theofilis et al. 2022; Castellana et al. 2021). MASLD diagnosis also requires at least one of the following cardiometabolic risk factors (Rinella et al. 2023): (1) overweight/obesity/central obesity: BMI ≥ 25 kg/m^2^, or WC > 94 cm (men) or > 80 cm (women); (2) hyperglycemia or diabetes: fasting blood glucose ≥ 5.6 mmol/L [100 mg/dL], 2‐h postprandial glucose ≥ 7.8 mmol/L [140 mg/dL], glycated hemoglobin (HbA1c) ≥ 5.7% [39 mmol/mol], prior diabetes diagnosis, or ongoing diabetes treatment; (3) hypertension: blood pressure ≥ 130/85 mmHg or use of antihypertensive medication; (4) elevated triglycerides: plasma TGs ≥ 1.70 mmol/L [150 mg/dL] or use of lipid‐lowering therapy; and (5) reduced high‐density lipoprotein cholesterol (HDL‐C): plasma HDL‐C ≤ 1.0 mmol/L [40 mg/dL] (men) and ≤ 1.3 mmol/L [50 mg/dL] (women), or ongoing lipid‐lowering treatment.

### 2.6 Covariates

Covariates included age (years), sex, race, education level, marital status, family poverty income ratio (PIR), smoking status, alcohol consumption, BMI, hypertension, diabetes, hyperlipidemia, antihypertensive drug usage, antihyperlipidemic drug usage, antidiabetic drug usage, and physical activity metabolic equivalent of task (PA‐MET).

Sex was classified as male or female. Race was classified as Mexican American, other Hispanic, non‐Hispanic White, non‐Hispanic Black, or other ethnicity. Education level was grouped as less than high school, high school or equivalent, or college and above. Marital status was classified as married or living with a partner, never married, or widowed/divorced/separated. PIR was divided into three levels: <1.3, 1.3–3.5, or > 3.5. Smoking status was determined by lifetime cigarette consumption and current smoking behavior and was classified as never smokers (no smoking history), former smokers (≥ 100 cigarettes but not currently smoking), or current smokers. Alcohol consumption was classified as never drinkers (no history of alcohol use), former drinkers (lifetime use > 12 drinks excluding the past year), and current drinkers (past‐year use > 12 drinks) based on the NHANES Alcohol Use Questionnaire (ALQ). BMI was calculated as weight (kg) divided by height (m) squared. Based on BMI values, weight status was categorized as normal weight (< 25 kg/m^2^), overweight (25–29.9 kg/m^2^), or obese (≥ 30 kg/m^2^). Hypertension was diagnosed if any of the following conditions were met: (1) systolic blood pressure ≥ 130 mmHg; (2) diastolic blood pressure ≥ 80 mmHg; or (3) prior diagnosis (Whelton et al. 2018). Diabetes was diagnosed if any of the following conditions were met: (1) prior diagnosis or current use of antidiabetic medications (pills or insulin); (2) HbA1c ≥ 6.5%; (3) fasting plasma glucose ≥ 126 mg/dL; (4) 2‐h plasma glucose ≥ 200 mg/dL (≥ 11.1 mmol/L) during an oral glucose tolerance test; or (5) random plasma glucose ≥ 200 mg/dL (≥ 11.1 mmol/L) (American Diabetes Association Professional Practice Committee 2025). Hyperlipidemia was diagnosed if any of the following conditions were met: (1) prior diagnosis; (2) total cholesterol ≥ 200 mg/dL; (3) triglycerides ≥ 150 mg/dL; (4) HDL‐C < 40 mg/dL (men) or < 50 mg/dL (women); or (5) low‐density lipoprotein cholesterol ≥ 130 mg/dL (Grundy et al. 2019). Physical activity (PA) was assessed using the World Health Organization's Global Physical Activity Questionnaire (GPAQ). Participants self‐reported the type and total duration of PA through the GPAQ. PA was converted into metabolic equivalent of task (MET) minutes per week for moderate‐to‐vigorous intensity activities. MET values vary by activity type, with NHANES providing recommended values for each activity. PA‐MET (minutes/week) was calculated as the sum of the products of each activity's MET value, weekly frequency, and session duration (Wei et al. 2024). Information on medication use (antihypertensive, antihyperlipidemic, and antidiabetic agents) was collected from self‐reports or prescription records, both of which have been considered highly reliable in similar studies for assessing disease status and medication use (Lai et al. 2025).

### 2.7 Statistical Analysis

All analyses were conducted using R software (version 4.3.1). We did not apply survey weights in the analyses, given that the study population was carefully selected and our primary aim was to assess internal comparisons within the sample. Normally distributed continuous variables are reported as the means ± standard deviations (SDs) and were compared using t tests; skewed variables are reported as medians with interquartile ranges (P25–P25) and were compared using Wilcoxon rank‐sum tests. Categorical variables are reported as frequencies (percentages) and were compared using *χ* ^2^ tests. To address multicollinearity and covariate selection, we assessed collinearity using variance inflation factors (VIFs), where, as a rule of thumb, values exceeding 5 indicate collinearity (Vatcheva et al. 2016). All the VIFs in our analysis were less than 5, allowing the inclusion of all the covariates. The FII and DII were analyzed as continuous variables to evaluate MASLD risk per 1‐SD increase using multivariable logistic regression with three models: Model 1 (unadjusted); Model 2 (adjusted for age, sex, race/ethnicity, education level, marital status, and PIR); and Model 3 (further adjusted for PA‐MET, smoking status, alcohol consumption, BMI, hypertension, hyperlipidemia, diabetes, antihypertensive drug usage, antihyperlipidemic drug usage and antidiabetic drug usage). P for trends across quartiles was computed. Restricted cubic spline (RCS) models were used to examine the nonlinear dose–response relationships between FII/DII and MASLD risk, using four knots at standard percentiles (5th, 35th, 65th, and 95th) to balance flexibility and stability, as is standard in epidemiological studies (Austin et al. 2022). Subgroup analyses, stratified by covariates (age [< 50 vs. ≥ 50 years], sex, race/ethnicity, education level, marital status, PIR, smoking status, alcohol consumption, hypertension, diabetes, or hyperlipidemia), were performed to exclude the stratifying variable itself, and heterogeneity was evaluated, with interaction tests identifying effect modifiers. With covariate missingness < 10% (Table S3), five sensitivity analyses were performed to assess the robustness of the results: (1) excluding hypertensive participants to evaluate the influence of comorbidities; (2) excluding participants with diabetes to minimize metabolic confounding; (3) using multiple imputation by chained equations (MICE) to generate five datasets, with pooled estimates using Rubin's rules; (4) limiting complete‐case analysis to participants with complete covariate data; and (5) performing reanalysis after extreme FII scores were trimmed (top/bottom 5%) to reduce outlier effects. Receiver operating characteristic (ROC) curves were constructed to compare the performance of the FII and DII for MASLD risk prediction using the area under the curve (AUC). Reclassification metrics (continuous net reclassification improvement [NRI] and integrated discrimination improvement [IDI]) were used to assess the FII's added value over the DII, with positive values (*p* < 0.05) indicating FII superiority. Two‐tailed *p* < 0.05 indicated statistical significance.

## 3 Results

### 3.1 Characteristics of Participants

This study ultimately included 25,067 participants, of whom 6708 met the MASLD diagnostic criteria. The general characteristics of the participants with and without MASLD are presented in Table S4. High FII scores were associated with significant differences in demographics, lifestyle, socioeconomic status, and metabolic conditions (all *p* < 0.001; Table 1). The mean age was 49.56 years, which was lower in Q4 (48.21 years) than in Q3 (50.27 years; *p* < 0.001). Q1 had a greater percentage of females (61.11%) compared to Q4 (37.04%; *p* < 0.001). The racial distribution differed, with non‐Hispanic Whites predominant in Q1 (54.28%) and other ethnicities more prevalent in Q4. Lower education levels (Q4: 33.59% vs. Q1: 18.06%) and lower income (PIR ≤ 1.3; Q4: 37.63% vs. Q1: 23.48%) were associated with higher FII scores. Lifestyle factors included reduced PA (Q1: 1680 vs. Q4: 1134 MET min/week), lower proportions of participants that were current smokers (Q1: 25.29% vs. Q4: 18.59%), and lower proportions of participants that were current drinkers (Q4: 56.49% vs. Q1: 77.24%) in higher FII quartiles (all *p* < 0.001). Obesity (BMI ≥ 30 kg/m^2^) was more prevalent in Q4 (40.27%) than in Q1 (33.14%), as were hypertension (Q4: 44.03% vs. Q1: 39.72%), diabetes (Q4: 22.20% vs. Q1: 16.55%), and hyperlipidemia (Q4: 73.96% vs. Q1: 70.88%, all *p* < 0.001). Antihypertensive drug usage (Q4: 32.41% vs. Q1: 28.44%) and antidiabetic drug usage (Q4: 12.84% vs. Q1: 9.52%) increased with FII score, but antihyperlipidemic drug usage was lower in Q4 than in Q1 to Q3 (Q4: 17.44% vs. Q1: 19.00%).

**TABLE 1:** Sample size and characteristics of the participants by FII scores.

	*n*	FII quartile				*p*
		Q1	Q2	Q3	Q4	
		< −8.92	−8.92 to −5.79	−5.78 to −3.69	> −3.69	
	25,067	6267	6266	6267	6267	
Age (years), mean (SD)	49.56 (17.82)	49.83 (16.13)	49.92 (17.49)	50.27 (18.45)	48.21 (19.01)	< 0.001
Gender (%)						
Male	12,912 (51.51)	2437 (38.89)	3016 (48.13)	3513 (56.06)	3946 (62.96)	< 0.001
Female	12,155 (48.49)	3830 (61.11)	3250 (51.87)	2754 (43.94)	2321 (37.04)	
Race (%)						
Mexican American	4349 (17.35)	853 (13.61)	1135 (18.11)	1203 (19.20)	1158 (18.48)	< 0.001
Other Hispanic	2181 (8.70)	461 (7.36)	526 (8.39)	570 (9.10)	624 (9.96)	
Non‐Hispanic White	11,034 (44.02)	3402 (54.28)	2883 (46.01)	2608 (41.61)	2141 (34.16)	
Non‐Hispanic Black	5115 (20.41)	801 (12.78)	1077 (17.19)	1357 (21.65)	1880 (30.00)	
Other Race	2388 (9.53)	750 (11.97)	645 (10.29)	529 (8.44)	464 (7.40)	
Educational level (%)						
Less than high school	6387 (25.51)	1131 (18.06)	1437 (22.94)	1718 (27.45)	2101 (33.59)	< 0.001
High school or equivalent	5801 (23.17)	1341 (21.41)	1440 (22.99)	1474 (23.55)	1546 (24.72)	
College or above	12,853 (51.33)	3792 (60.54)	3387 (54.07)	3067 (49.00)	2607 (41.69)	
Marital status (%)						
Married and a partner	4266 (17.17)	922 (14.80)	911 (14.64)	1117 (18.02)	1316 (21.25)	< 0.001
Never married	15,263 (61.44)	4080 (65.51)	4101 (65.91)	3663 (59.08)	3419 (55.20)	
Widowed, divorced or separated	5315 (21.39)	1226 (19.69)	1210 (19.45)	1420 (22.90)	1459 (23.56)	
Poverty to income ratio (%)						
< 1.3	6722 (29.43)	1358 (23.48)	1522 (26.70)	1704 (30.01)	2138 (37.63)	< 0.001
1.3–3.5	8871 (38.83)	2057 (35.57)	2118 (37.16)	2375 (41.83)	2321 (40.85)	
> 3.5	7250 (31.74)	2368 (40.95)	2060 (36.14)	1599 (28.16)	1223 (21.52)	
Body mass index (%)						
< 25	7306 (29.15)	1992 (31.79)	1858 (29.65)	1706 (27.22)	1750 (27.92)	< 0.001
25–29.9	8562 (34.16)	2198 (35.07)	2197 (35.06)	2174 (34.69)	1993 (31.80)	
≥ 30	9199 (36.70)	2077 (33.14)	2211 (35.29)	2387 (38.09)	2524 (40.27)	
Smoking status (%)						
Never	13,546 (54.08)	2859 (45.64)	3271 (52.22)	3628 (57.94)	3788 (60.51)	< 0.001
Former	6368 (25.42)	1821 (29.07)	1698 (27.11)	1541 (24.61)	1308 (20.89)	
Current	5136 (20.50)	1584 (25.29)	1295 (20.67)	1093 (17.45)	1164 (18.59)	
Alcohol consumption (%)						
Never	3071 (12.82)	427 (7.12)	580 (9.67)	874 (14.54)	1190 (20.01)	< 0.001
Former	4700 (19.62)	937 (15.63)	1050 (17.50)	1315 (21.87)	1398 (23.50)	
Current	16,181 (67.56)	4629 (77.24)	4369 (72.83)	3823 (63.59)	3360 (56.49)	
Physical activity, MET*min/week, median(IQR)	1437.13 (480.00–4080.00)	1680.00 (560.00–4800.00)	1440.00 (480.00–4320.00)	1200.00 (400.00–3780.00)	1134.00 (360.00–3780.00)	< 0.001
Hypertension (%)						
No	14,500 (57.85)	3778 (60.28)	3697 (59.01)	3518 (56.15)	3507 (55.97)	< 0.001
Yes	10,563 (42.15)	2489 (39.72)	2568 (40.99)	2747 (43.85)	2759 (44.03)	
Hyperlipidemia (%)						
No	6763 (26.98)	1825 (29.12)	1684 (26.88)	1622 (25.88)	1632 (26.04)	< 0.001
Yes	18,304 (73.02)	4442 (70.88)	4582 (73.12)	4645 (74.12)	4635 (73.96)	
Diabetes (%)						
No	19,621 (80.40)	5165 (83.45)	4991 (81.61)	4766 (78.66)	4699 (77.80)	< 0.001
Yes	4783 (19.60)	1024 (16.55)	1125 (18.39)	1293 (21.34)	1341 (22.20)	
Antihypertensive drug usage						
No	17,288 (69.02)	4481 (71.56)	4352 (69.48)	4224 (67.47)	4231 (67.59)	< 0.001
Yes	7759 (30.98)	1781 (28.44)	1912 (30.52)	2037 (32.53)	2029 (32.41)	
Antihyperlipidemic drug usage						
No	20,377 (81.36)	5072 (81.00)	5066 (80.87)	5071 (80.99)	5168 (82.56)	0.047
Yes	4670 (18.64)	1190 (19.00)	1198 (19.13)	1190 (19.01)	1092 (17.44)	
Antidiabetic drug usage						
No	22,247 (88.82)	5666 (90.48)	5614 (89.62)	5511 (88.02)	5456 (87.16)	< 0.001
Yes	2800 (11.18)	596 (9.52)	650 (10.38)	750 (11.98)	804 (12.84)	

### 3.2 Association Between the FII and MASLD

Multivariable logistic regression analyses revealed a significant positive association between FII scores and MASLD risk (Table 2). In the unadjusted model (Model 1), a 1‐SD increase in FII score was associated with a 16.7% higher risk of MASLD (OR = 1.167, 95% CI: 1.127–1.208; *p* < 0.00001). In Model 2, adjusted for demographic variables, a 1‐SD increase in FII score was associated with an 18.9% increase in MASLD risk (OR = 1.189, 95% CI: 1.143–1.236; *p* < 0.00001). In Model 3, which was further adjusted for lifestyle factors, comorbidities, and medication use, the risk remained elevated (OR = 1.079, 95% CI: 1.025–1.137; *p* = 0.00402). In Model 3, when Q1 was used as the reference, MASLD risk was significantly greater for Q2 (OR = 1.141, 95% CI: 1.008–1.293; *p* = 0.03771), Q3 (OR = 1.162, 95% CI: 1.023–1.321; *p* = 0.02122), and Q4 (OR = 1.308, 95% CI: 1.143–1.496; *p* = 0.00009), with a significant dose–response trend (P for trend = 0.00016). The DII was also positively associated with MASLD risk (OR = 1.091, 95% CI: 1.047–1.138; *p* = 0.00004; Table S5). Quartile analysis of DII revealed significantly higher MASLD risk in Q2, Q3, and Q4 than in Q1 (all *p* < 0.05), with a significant trend (P for trend = 0.0009). However, the differences in risk across DII quartiles in Model 3 were smaller than those for the FII.

**TABLE 2:** Association between FII and MASLD by logistic regression.

Exposure	Model 1	Model 2	Model 3
	OR (95% CI), *p*	OR (95% CI), *p*	OR (95% CI), *p*
FII *Z*‐score	1.167 (1.127, 1.208), < 0.00001	1.189 (1.143, 1.236), < 0.00001	1.079 (1.025, 1.137), 0.00402
FII quartile			
Q1	1	1	1
Q2	1.172 (1.080, 1.272), 0.00014	1.166 (1.068, 1.272), 0.00058	1.141 (1.008, 1.293), 0.03771
Q3	1.341 (1.237, 1.454), < 0.00001	1.362 (1.248, 1.487), < 0.00001	1.162 (1.023, 1.321), 0.02122
Q4	1.437 (1.326, 1.556), < 0.00001	1.538 (1.406, 1.682), < 0.00001	1.308 (1.143, 1.496), 0.00009
*p* for trend	< 0.00001	< 0.00001	0.00016

*Note:* Model 1: Nonadjusted. Model 2: Adjusted for age, gender, race, educational level, marital status, PIR. Model 3: Adjusted for age, gender, race, educational level, marital status, PIR, BMI, smoking status, alcohol consumption, hypertension, hyperlipidemia, diabetes, physical activity, antihypertensive drug usage, antihyperlipidemic drug usage, and antidiabetic drug usage.

### 3.3 Dose–Response Analysis

**FIGURE 2:**
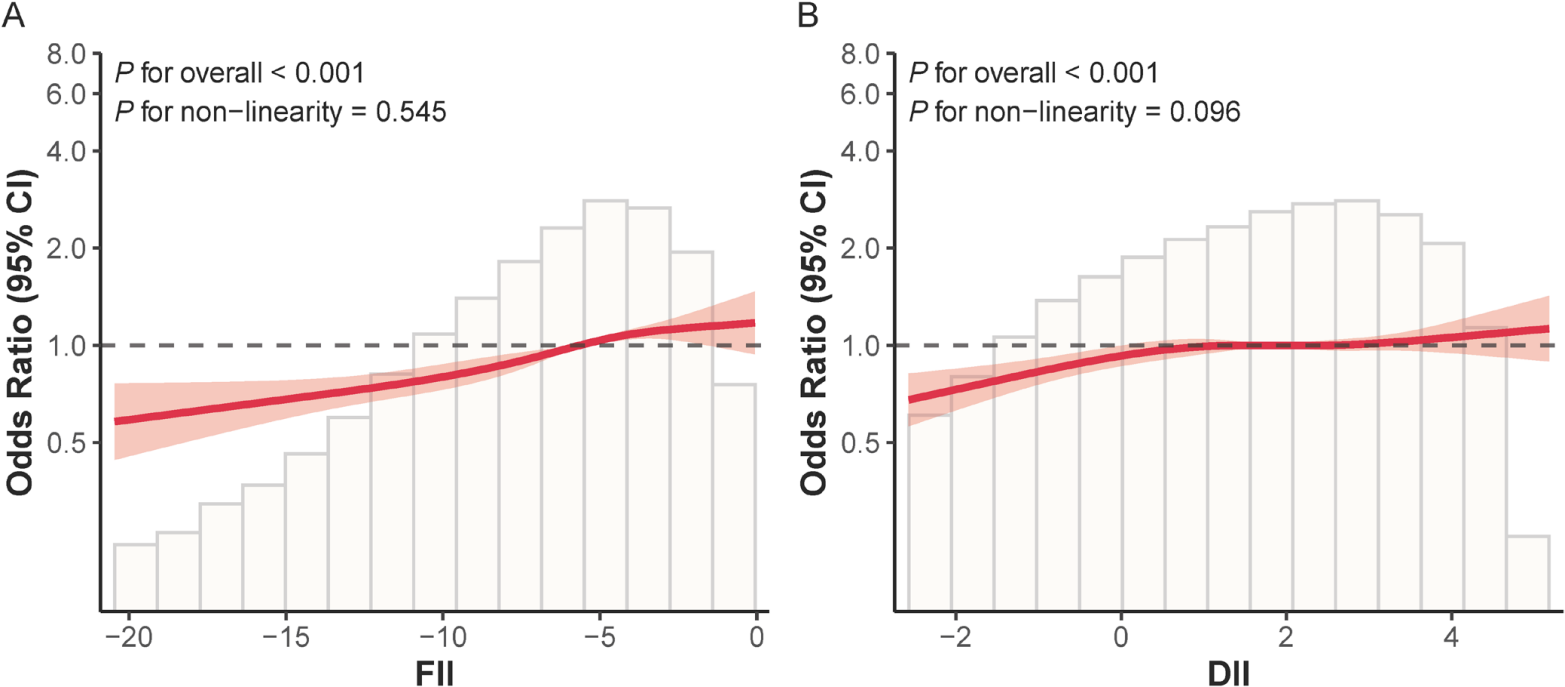
Associations of FII and DII with MASLD evaluated by RCS after covariate adjustment.

RCS analysis, adjusted for all Model 3 covariates, was conducted to evaluate potential nonlinear associations. RCS analysis confirmed a linear dose–response relationship between higher FII scores and increased MASLD risk (*p* < 0.001; *p*‐nonlinearity = 0.545). These findings indicate that the MASLD risk increased progressively with increasing FII scores, without threshold effects (Figure 2A). RCS analysis of the DII revealed a similar linear dose–response relationship with MASLD risk (*p* < 0.001; *p*‐nonlinearity = 0.096; Figure 2B).

### 3.4 Subgroup Analysis

**FIGURE 3:**
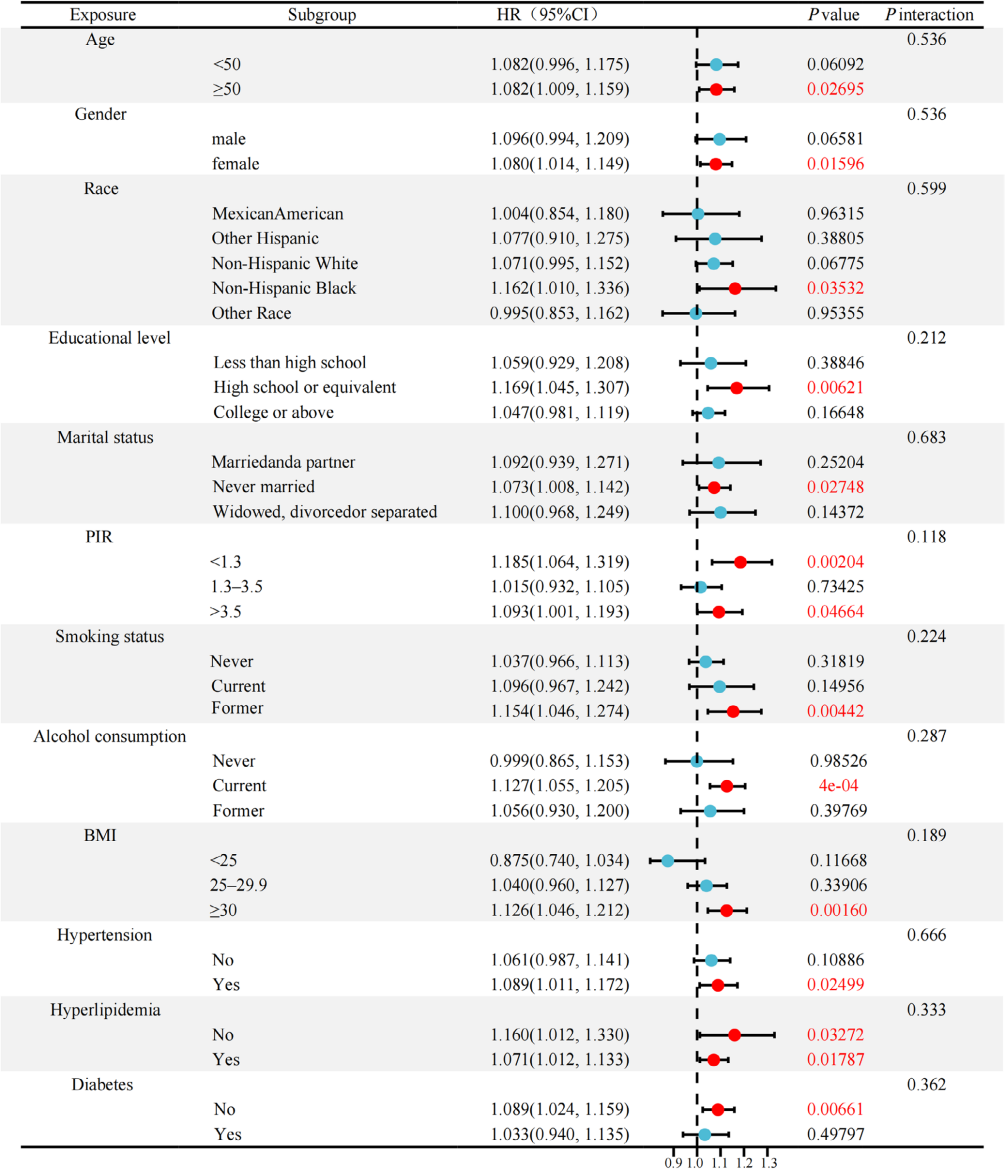
Subgroup analysis and forest plot visualization of the association between the FII and MASLD.

Subgroup analyses were performed to assess whether demographic and clinical characteristics modified the association between the FII and MASLD (Figure 3). Positive associations between FII score and MASLD risk were observed among participants aged ≥ 50 years, women, non‐Hispanic Black individuals, individuals with high school or equivalent education, unmarried individuals, those with high income (PIR > 3.5), individuals who were former smokers, individuals who were current drinkers, individuals classified as obese, individuals with hypertension, individuals with or without hyperlipidemia, and individuals without diabetes (all *p* < 0.05). No significant interactions were observed across the subgroups (all *p*‐interaction > 0.05). This finding indicates that the positive association between the FII and MASLD was consistent across diverse population characteristics, with no subgroup significantly altering its strength or direction.

### 3.5 Sensitivity Analyses

To assess the robustness of the association between the FII and MASLD, we conducted five sensitivity analyses. Excluding participants with diabetes, a significant association between FII score and MASLD risk was maintained (OR = 1.125, 95% CI: 1.064–1.190; *p* = 0.00003; Table S6). This suggests that diabetes was not a dominant confounder. Excluding participants with hypertension preserved the association's direction, with a slightly reduced but significant OR (OR = 1.110, 95% CI: 1.036–1.189; *p* = 0.00288; Table S7). This finding indicates that the association was not dependent on hypertension. Complete‐case analysis, excluding participants with missing covariates, yielded results consistent with those of the primary analysis (OR = 1.082 vs. 1.079; Table S8). This suggests that missing data had minimal impact on the findings. Trimming the top and bottom 5% of the FII scores maintained a positive association (OR = 1.083, 95% CI: 1.025–1.144; *p* = 0.00452; Table S9). This confirms that outliers did not drive the results. Multiple imputation created five complete datasets, with pooled analyses yielding an OR consistent with that of the primary analysis (OR = 1.081, 95% CI: 1.037–1.128; *p* = 0.00027; Table S10). This finding indicates that imputation did not alter the statistical inferences. These sensitivity analyses collectively strengthened the primary model's findings, confirming a robust positive association between FII score and MASLD risk.

### 3.6 Comparison of FII and DII Performance Using the ROC, NRI, and IDI

**FIGURE 4:**
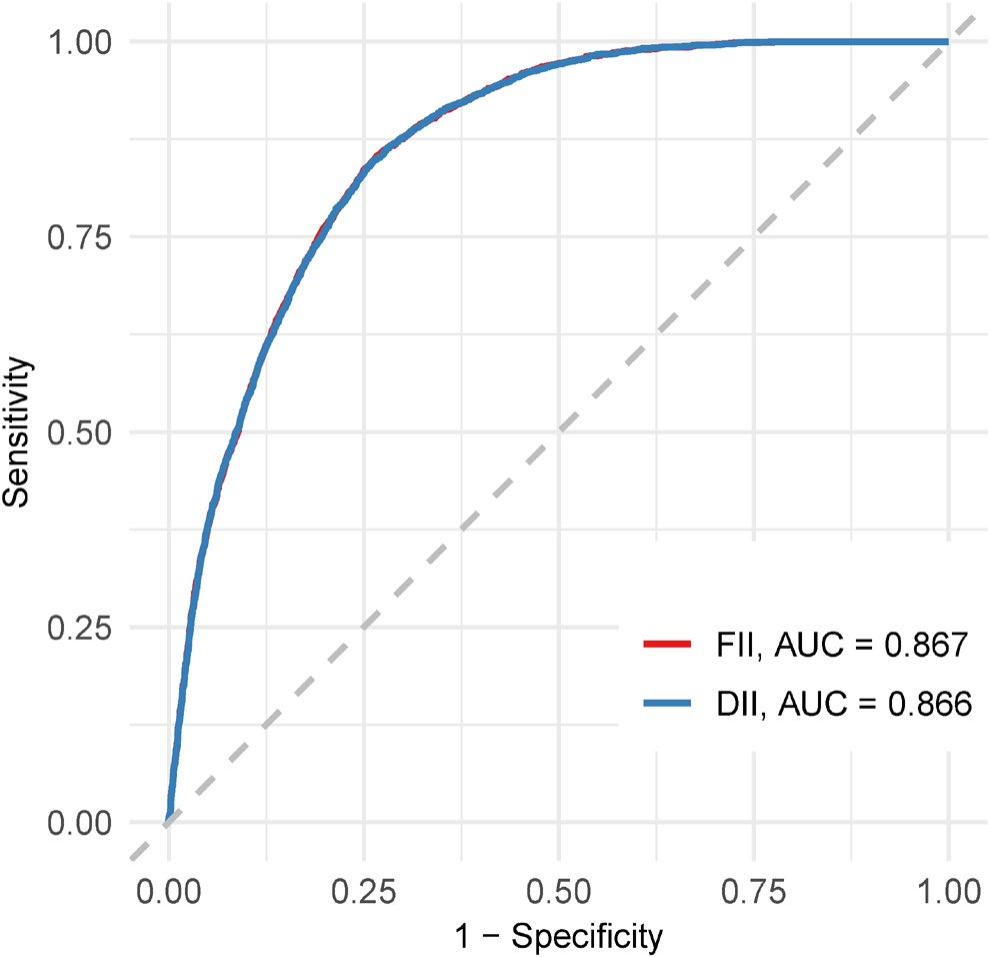
Comparison of FII and DII in predicting MASLD risk using ROC curves.

ROC curve analysis (Figure 4, Table 3) revealed AUC values for the FII and DII for predicting MASLD risk of 0.867 (95% CI: 0.861–0.872) and 0.866 (95% CI: 0.860–0.872), respectively, with no significant difference (*p* = 0.272). The FII exhibited slightly higher sensitivity (0.854 vs. 0.848, respectively) but lower specificity (0.733 vs. 0.738, respectively) than those of the DII. NRI and IDI analyses (Table 4) revealed that the FII outperformed the DII, with an NRI of 0.0556 (95% CI: 0.0204–0.0908; *p* = 0.002) and an IDI of 0.0005 (95% CI: 0.0001–0.0008; *p* = 0.015). These results indicate that compared with the DII, the FII improved risk reclassification and model discrimination.

**TABLE 3:** Predictive performance of FII and DII for MASLD risk using ROC analysis.

Measure	Best threshold	Sensitivity	Specificity	AUC (95% CI)	*p*	*p* for difference
DII	0.214	0.848	0.738	0.866 (0.860–0.872)	< 0.001	Reference
FII	0.21	0.854	0.733	0.867 (0.861–0.872)	< 0.001	0.272

**TABLE 4:** Comparison of FII and DII for MASLD risk prediction using NRI and IDI.

Models	NRI (95% CI)	*p*	IDI (95% CI)	*p*
DII	Reference	Reference	Reference	Reference
FII	0.0556 (0.0204, 0.0908)	0.002	0.0005 (0.0001, 0.0008)	0.015

## 4 Discussion

This study confirms a significant association between higher FII scores and increased MASLD risk in a large U.S. cohort (NHANES, *n* = 25,067). Our findings revealed a linear dose–response relationship (*p*‐trend < 0.001). A 1‐SD increase in FII score was associated with a 7.9% increased MASLD risk (OR = 1.079, 95% CI: 1.025–1.137) after adjustment for demographics, lifestyle, comorbidities, and medication use. Compared with the DII, the FII demonstrates comparable diagnostic accuracy (FII AUC = 0.867 vs. DII AUC = 0.866) but significantly enhances risk reclassification by 5.56% (NRI = 0.0556; *p* = 0.002) and discrimination by 0.05% (IDI = 0.0005; *p* = 0.015). These findings indicate greater sensitivity to dietary inflammatory potential and establish the FII as a valuable tool for evaluating dietary contributions to MASLD, providing a basis for targeted nutritional interventions to reduce disease risk.

Our findings align with previous research linking proinflammatory diets to MASLD. A meta‐analysis of 242,006 participants revealed a 63% higher NAFLD risk with elevated DII scores (OR = 1.63, 95% CI: 1.32–2.01) (Zhao et al. 2024). Pediatric studies similarly reported a 2.6‐fold increased risk of advanced hepatic steatosis per unit increase in DII (Amiri et al. 2025). However, in contrast to prior studies reporting nonlinear associations between DII and NAFLD/MAFLD—such as Zhang et al. 2023 (nonlinear DII‐NAFLD with a turning point at 1.80) and Yan et al. (2024) (nonlinear DII‐MAFLD with a turning point at 3.06)—our analysis revealed a linear dose–response curve (*p*‐nonlinearity = 0.545), suggesting that the FII better captures dietary inflammatory variations across the full spectrum. This advantage arises from the FII's portion‐size standardization, which adjusts for intake levels of pro‐ and anti‐inflammatory components, addressing the DII's limitations in classifying foods with minimal anti‐inflammatory content, such as cucumbers (vitamin C: ~4.8 g/100 g) (Wang et al. 2025). For example, the FII emphasizes saturated fats and flavonoids, which influence hepatic lipid peroxidation and insulin resistance—key MASLD mechanisms (Rosqvist et al. 2019; Kozłowska 2025). The exclusion of foods such as garlic and onions, due to inconsistent NHANES dietary recall data, enhances FII reproducibility but may limit its scope (Israelsen et al. 2024). ROC analysis (FII AUC = 0.867 vs. DII AUC = 0.866; *p* = 0.272) and significant improvements in the NRI/IDI (Table 4) confirm that the FII has a slight advantage in terms of predictive accuracy for MASLD risk stratification.

The association between the FII and MASLD is biologically plausible and is driven by specific dietary components. Proinflammatory saturated fats and refined carbohydrates promote hepatic lipid peroxidation and NF‐κB‐mediated cytokine release, exacerbating steatosis (Gao et al. 2022; Chen et al. 2020; Feng et al. 2022). Conversely, anti‐inflammatory flavonoids (e.g., flavanones) (Bell et al. 2024) and dietary fiber (Akhgarjand et al. 2024; Chen et al. 2024), prioritized in the FII, reduce serum cholesterol and improve insulin sensitivity, mitigating MASLD progression. These effects align with FII's design, which weights components by their inflammatory impact (TIS), previously validated against CRP (Wang et al. 2025).

The superior performance of the FII over the DII in predicting MASLD risk (NRI = 0.0556; *p* = 0.002; Table 4) likely stems from its comprehensive inclusion of bioactive fatty acids, including MUFAs and PUFAs such as linoleic acid (18:2), α‐linolenic acid (ALA, 18:3), arachidonic acid (20:4), eicosapentaenoic acid (EPA, 20:5 n‐3), docosahexaenoic acid (DHA, 22:6 n‐3), and docosapentaenoic acid (DPA, 22:5 n‐3). These fatty acids are pivotal in both the pathogenesis and prevention of MASLD and related chronic metabolic and inflammatory disorders. A meta‐analysis revealed that omega‐3 PUFAs (EPA and DHA) significantly reduce liver fat, transaminases, and body composition markers in patients with MASLD, highlighting the protective role of these compounds (Moore et al. 2024). Clinical trials further demonstrate that EPA and DHA supplementation alleviates hepatic steatosis and inflammation while improving insulin sensitivity and reducing cardiovascular risk, key MASLD comorbidities (Banaszak et al. 2024). Replacing saturated fatty acids with MUFAs and PUFAs, such as linoleic acid and ALA, decreases type 2 diabetes risk by enhancing glycemic control and mitigating adipose tissue inflammation, processes closely tied to MASLD progression (Sivri and Akdevelioğlu 2025). Moreover, MUFAs and omega‐3 PUFAs (ALA, EPA, DHA, and DPA) improve cardiovascular outcomes, with DPA showing particular efficacy in reducing coronary event risk (Del Gobbo et al. 2016). These fatty acids modulate critical MASLD pathways, including hepatic lipid peroxidation, NF‐κB signaling, and peroxisome proliferator‐activated receptor‐gamma (PPAR‐γ) activity (Spooner and Jump 2023). By focusing on these components, FII enhances sensitivity to dietary patterns that reduce MASLD risk, serving as a precise tool for clinical dietary interventions. Clinicians should advocate diets rich in MUFAs and omega‐3 PUFAs (e.g., fatty fish, flaxseed, and walnuts) and minimize saturated fat intake.

Several limitations should be considered. First, the cross‐sectional design of NHANES data limits causal inference between the FII and MASLD. Second, MASLD diagnosis relies on the FLI (≥ 60), which, while validated (Bedogni et al. 2006), may introduce diagnostic imprecision compared with liver biopsy or MRI. Third, 24‐h dietary recalls may introduce recall bias and capture only short‐term intake, potentially affecting FII accuracy and relevance to chronic MASLD. Fourth, excluding anti‐inflammatory foods such as garlic and onions due to inconsistent NHANES reporting may lead to the underestimation of FII sensitivity. Fifth, this study's focus on U.S. adults limits generalizability to populations with distinct dietary patterns or demographic profiles. Sixth, as a novel tool introduced in 2024, the FII's validation is limited to Mediterranean diet components, potentially restricting confidence in its broader applicability. Finally, due to data constraints, comparisons with other indices (e.g., E‐DII and INFLAME) were not feasible, limiting the benchmarking of FII performance. To address these limitations, future studies should use longitudinal designs, combine FFQs or biomarkers (e.g., fatty acids and liver enzymes) with recalls, and validate the FII in diverse populations to enhance causality and applicability.

Our findings suggest that the FII may serve as a potential tool for informing personalized nutritional strategies aimed at MASLD prevention, although our results are based on preliminary correlational analyses. For instance, reducing the intake of high‐FII foods (e.g., red meat, refined sugars) and increasing the consumption of low‐FII foods (e.g., fiber‐rich vegetables, flavonoid‐rich fruits) could decrease the risk of MASLD. Future studies should validate the FII against inflammatory biomarkers, compare it with E‐DII and INFLAME in diverse cohorts, and assess FII‐guided interventions in randomized controlled trials. These efforts will increase the utility of the FII as a practical tool for managing MASLD and other inflammatory conditions.

## 5 Conclusion

This study revealed a linear positive correlation between the FII and MASLD, deepening our understanding of the role of dietary inflammation in MASLD pathogenesis. We recommend reducing high‐FII foods (e.g., red meat, refined sugars) and increasing low‐FII foods (e.g., vegetables, fruits) to lower MASLD risk. As an enhanced version of the DII, the FII's 5.56% improvement in risk reclassification (NRI = 0.0556, *p* = 0.002) and 0.05% enhancement in discrimination (IDI = 0.0005, *p* = 0.015) highlight its potential for personalized dietary strategies. The incorporation of the FII into personalized nutritional strategies may open new avenues for alleviating the global burden of MASLD and other inflammation‐related diseases.

## Author Contributions

**Qingwan Yang:** conceptualization (lead), data curation (equal), formal analysis (equal), supervision (lead), writing – original draft (lead). **Xin Cai:** conceptualization (equal), data curation (lead), investigation (equal), methodology (equal), software (lead), writing – review and editing (equal). **Zhenghua Xiao:** conceptualization (equal), funding acquisition (lead), project administration (lead), supervision (lead), writing – review and editing (equal).

## Consent

The authors have nothing to report.

## Conflicts of Interest

The authors declare no conflicts of interest.

## Supporting information


**Table S1:** fsn370865‐sup‐0001‐TablesS1‐S10.docx.

## Data Availability

The original data presented in the study are openly available at the National Health and Nutrition Examination Survey at https://wwwn.cdc.gov/nchs/nhanes/.
