# Supplementary material for: Higher Food Inflammation Index Is Linearly Associated With Higher Risk of MASLD: A Cross‐Sectional Study Based on the NHANES (1999–2020)
Source: Food Sci Nutr. 2025 Sep 2;13(9):e70865. doi: 10.1002/fsn3.70865 (PMC12402601; doi:10.1002/fsn3.70865)
Supplement: Supplementary file 1 — Table S1: fsn370865‐sup‐0001‐TablesS1‐S10.docx. [file FSN3-13-e70865-s001.docx]

**Table S1 Food parameters included in the DII, inflammatory effect scores, and intake values**

| Food parameter | Inflammatory effect score | Global daily average intake (units/d) | SD |
| --- | --- | --- | --- |
| Alcohol (g) | −0·278 | 13·98 | 3·72 |
| Vitamin B12 (μg) | 0·106 | 5·15 | 2·70 |
| Vitamin B6 (mg) | −0·365 | 1·47 | 0·74 |
| β-Carotene (μg) | −0·584 | 3718 | 1720 |
| Caffeine (g) | −0·110 | 8·05 | 6·67 |
| Carbohydrate (g) | 0·097 | 272·2 | 40·0 |
| Cholesterol (mg) | 0·110 | 279·4 | 51·2 |
| Energy (kcal) | 0·180 | 2056 | 338 |
| Eugenol (mg) | −0·140 | 0·01 | 0·08 |
| Total fat (g) | 0·298 | 71·4 | 19·4 |
| Fibre (g) | −0·663 | 18·8 | 4·9 |
| Folic acid (μg) | −0·190 | 273·0 | 70·7 |
| Garlic (g) | −0·412 | 4·35 | 2·90 |
| Ginger (g) | −0·453 | 59·0 | 63·2 |
| Fe (mg) | 0·032 | 13·35 | 3·71 |
| Mg (mg) | −0·484 | 310·1 | 139·4 |
| MUFA (g) | −0·009 | 27·0 | 6·1 |
| Niacin (mg) | −0·246 | 25·90 | 11·77 |
| n-3 Fatty acids (g) | −0·436 | 1·06 | 1·06 |
| n-6 Fatty acids (g) | −0·159 | 10·80 | 7·50 |
| Onion (g) | −0·301 | 35·9 | 18·4 |
| Protein (g) | 0·021 | 79·4 | 13·9 |
| PUFA (g) | −0·337 | 13·88 | 3·76 |
| Riboflavin (mg) | −0·068 | 1·70 | 0·79 |
| Saffron (g) | −0·140 | 0·37 | 1·78 |
| Saturated fat (g) | 0·373 | 28·6 | 8·0 |
| Se (μg) | −0·191 | 67·0 | 25·1 |
| Thiamin (mg) | −0·098 | 1·70 | 0·66 |
| Trans fat (g) | 0·229 | 3·15 | 3·75 |
| Turmeric (mg) | −0·785 | 533·6 | 754·3 |
| Vitamin A (RE) | −0·401 | 983·9 | 518·6 |
| Vitamin C (mg) | −0·424 | 118·2 | 43·46 |
| Vitamin D (μg) | −0·446 | 6·26 | 2·21 |
| Vitamin E (mg) | −0·419 | 8·73 | 1·49 |
| Zn (mg) | −0·313 | 9·84 | 2·19 |
| Green/black tea (g) | −0·536 | 1·69 | 1·53 |
| Flavan-3-ol (mg) | −0·415 | 95·8 | 85·9 |
| Flavones (mg) | −0·616 | 1·55 | 0·07 |
| Flavonols (mg) | −0·467 | 17·70 | 6·79 |
| Flavonones (mg) | −0·250 | 11·70 | 3·82 |
| Anthocyanidins (mg) | −0·131 | 18·05 | 21·14 |
| Isoflavones (mg) | −0·593 | 1·20 | 0·20 |
| Pepper (g) | −0·131 | 10·00 | 7·07 |
| Thyme/oregano (mg) | −0·102 | 0·33 | 0·99 |
| Rosemary (mg) | −0·013 | 1·00 | 15·00 |

**Table S2 The total inflammation score (TIS) and nutrient recommended values (NRVs) of nutrient components.**

| Name | Unit name | TIS | NRVs |
| --- | --- | --- | --- |
| Protein | g | 0.021 | 56 |
| Total lipid (fat) | g | 0.298 | 77 |
| Carbohydrate, by difference | g | 0.097 | 130 |
| Energy | kcal | 0.18 | 2200 |
| Alcohol, ethyl | g | -0.278 | 5.15 |
| Caffeine | mg | -0.11 | 8.05 |
| Fiber, total dietary | g | -0.663 | 31 |
| Iron, Fe | mg | 0.032 | 8 |
| Magnesium, Mg | mg | -0.484 | 420 |
| Zinc, Zn | mg | -0.313 | 11 |
| Selenium, Se | μg | -0.191 | 67 |
| Vitamin A, RAE | μg | -0.401 | 900 |
| Carotene, alpha | μg | -0.584 | 3718 |
| Vitamin E (alpha-tocopherol) | mg | -0.419 | 15 |
| Vitamin D (D2 + D3) | μg | -0.446 | 15 |
| Vitamin C, total ascorbic acid | mg | -0.424 | 90 |
| Thiamin | mg | -0.098 | 1.2 |
| Riboflavin | mg | -0.068 | 1.3 |
| Niacin | mg | -0.2462 | 16 |
| Vitamin B-6 | mg | -0.365 | 1.3 |
| Vitamin B-12 | μg | 0.106 | 2.4 |
| Folic acid | μg | -0.19 | 400 |
| Vitamin E, added | mg | -0.419 | 15 |
| Vitamin B-12, added | μg | 0.106 | 2.4 |
| Cholesterol | mg | 0.11 | 279.4 |
| Fatty acids, total saturated | g | 0.373 | 22 |
| PUFA 18:2 | g | -0.159 | 10.8 |
| PUFA 18:3 | g | -0.436 | 1.06 |
| PUFA 20:4 | g | -0.159 | 10.8 |
| PUFA 22:6 n-3 (DHA) | g | -0.436 | 1.06 |
| PUFA 20:5 n-3 (EPA) | g | -0.436 | 1.06 |
| PUFA 22:5 n-3 (DPA) | g | -0.436 | 1.06 |
| Fatty acids, total monounsaturated | g | -0.009 | 27 |
| Fatty acids, total polyunsaturated | g | -0.337 | 13.88 |
| Flavan-3-ols | mg | -0.415 | 95.8 |
| Flavanones | mg | -0.25 | 11.7 |
| Flavones | mg | -0.616 | 1.55 |
| Flavonols | mg | -0.467 | 17.7 |
| Isoflavones | mg | -0.593 | 1.2 |

**Table S3 Number of participants with complete data**

| Complete Data | Number |
| --- | --- |
| Education | 26 |
| Marital status | 223 |
| PIR | 2224 |
| Smoker | 17 |
| Alcohol consumption | 1115 |
| Hypertension | 4 |
| Diabetes | 663 |
| Anti-hypertensive drug usage | 20 |
| Anti-hyperlipidemic drug usage | 20 |
| Anti-diabetic drug usage | 20 |
| Physical activity_MET | 653 |

**Table S4 Sample size and characteristics of MASLD and non-MASLD participants**

|  | Overall | MASLD | Non-MASLD | *P*-value |
| --- | --- | --- | --- | --- |
|  | 25067 | 18359 | 6708 |  |
| FII | -5.79(-8.92--3.69) | -5.94(-9.14--3.79) | -5.34(-8.27--3.46) | <0.001 |
| DII | 1.78 (0.26-2.99) | 1.75 (0.19-2.96) | 1.88 (0.45-3.06) | <0.001 |
| Age（years）, mean (SD) | 49.56 (17.82) | 47.84 (17.97) | 54.24 (16.51) | <0.001 |
| Gender (%) |  |  |  | <0.001 |
| Male | 12912 (51.51) | 9620 (52.40) | 3292 (49.08) |  |
| Female | 12155 (48.49) | 8739 (47.60) | 3416 (50.92) |  |
| Race (%) |  |  |  | <0.001 |
| Mexican American | 4349 (17.35) | 3118 (16.98) | 1231 (18.35) |  |
| Other Hispanic | 2181 (8.70) | 1619 (8.82) | 562 (8.38) |  |
| Non-Hispanic White | 11034 (44.02) | 7973 (43.43) | 3061 (45.63) |  |
| Non-Hispanic Black | 5115 (20.41) | 3700 (20.15) | 1415 (21.09) |  |
| Other Race | 2388 (9.53) | 1949 (10.62) | 439 (6.54) |  |
| Educational level (%) |  |  |  | <0.001 |
| Less than high school | 6387 (25.51) | 4494 (24.51) | 1893 (28.22) |  |
| High school or equivalent | 5801 (23.17) | 4241 (23.13) | 1560 (23.26) |  |
| College or above | 12853 (51.33) | 9599 (52.36) | 3254 (48.52) |  |
| Marital status (%) |  |  |  | <0.001 |
| Married and a partner | 4266 (17.17) | 3501 (19.24) | 765 (11.51) |  |
| Never married | 15263 (61.44) | 10906 (59.93) | 4357 (65.55) |  |
| Widowed, divorced or separated | 5315 (21.39) | 3790 (20.83) | 1525 (22.94) |  |
| Poverty to income ratio (%) |  |  |  | 0.124 |
| <1.3 | 6722 (29.43) | 4904 (29.29) | 1818 (29.81) |  |
| 1.3 – 3.5 | 8871 (38.83) | 6463 (38.60) | 2408 (39.49) |  |
| > 3.5 | 7250 (31.74) | 5378 (32.12) | 1872 (30.70) |  |
| Body mass index (%) |  |  |  | <0.001 |
| <25 | 7306 (29.15) | 7223 (39.34) | 83 (1.24) |  |
| 25 – 29.9 | 8562 (34.16) | 6973 (37.98) | 1589 (23.69) |  |
| ≥30 | 9199 (36.70) | 4163 (22.68) | 5036 (75.07) |  |
| Smoking status (%) |  |  |  | <0.001 |
| Never | 13546 (54.08) | 9722 (53.00) | 3824 (57.02) | ) |
| Former | 6368 (25.42) | 4351 (23.72) | 2017 (30.08) |  |
| Current | 5136 (20.50) | 4271 (23.28) | 865 (12.90) |  |
| Alcohol consumption (%) |  |  |  | <0.001 |
| Never | 3071 (12.82) | 1874 (10.62) | 1197 (18.99) |  |
| Former | 4700 (19.62) | 2642 (14.97) | 2058 (32.65) |  |
| Current | 16181 (67.56) | 13133 (74.41) | 3048 (48.36) |  |
| Physical activity, MET*min/week, median(IQR) | 1437.13 (480.00-4080.00) | 1440.00 (480.00-4320.00) | 1200.00 (404.63-3600.00) | <0.001 |
| Hypertension (%) |  |  |  | <0.001 |
| No | 14500 (57.85) | 11651 (63.48) | 2849 (42.47) |  |
| Yes | 10563 (42.15) | 6704 (36.52) | 3859 (57.53) |  |
| Hyperlipidemia (%) |  |  |  | <0.001 |
| No | 6763 (26.98) | 5876 (32.01) | 887 (13.22) |  |
| Yes | 18304 (73.02) | 12483 (67.99) | 5821 (86.78) |  |
| Diabetes (%) |  |  |  | <0.001 |
| No | 19621 (80.40) | 15247 (85.36) | 4374 (66.87) |  |
| Yes | 4783 (19.60) | 2616 (14.64) | 2167 (33.13) |  |
| Anti-hypertensive drug usage |  |  |  | <0.001 |
| No | 17288 (69.02) | 13657 (74.44) | 3631 (54.19) |  |
| Yes | 7759 (30.98) | 4689 (25.56) | 3070 (45.81) |  |
| Anti-hyperlipidemic drug usage |  |  |  | <0.001 |
| No | 20377 (81.36) | 15454 (84.24) | 4923 (73.47) |  |
| Yes | 4670 (18.64) | 2892 (15.76) | 1778 (26.53) |  |
| Anti-diabetic drug usage |  |  |  | <0.001 |
| No | 22247 (88.82) | 16847 (91.83) | 5400 (80.58) |  |
| Yes | 2800 (11.18) | 1499 (8.17) | 1301 (19.42) |  |

**Table S5 Multivariable Logistic Regression Analysis of the Association Between DII and MASLD Risk**

| Exposure | Model 1 | Model 2 | Model 3 | |
| --- | --- | --- | --- | --- |
| DII Z-score | 1.098 (1.067, 1.129) <0.00001 | 1.111 (1.076, 1.147) <0.00001 | 1.091 (1.047, 1.138) 0.00004 | |
| DII quartile |  |  |  | |
| Q1 | 1 | 1 | 1 | |
| Q2 | 1.197 (1.105, 1.297) 0.00001 | 1.204 (1.105, 1.312) 0.00002 | 1.229 (1.101, 1.372) 0.00024 | |
| Q3 | 1.165 (1.075, 1.263) 0.00019 | 1.192 (1.092, 1.301) 0.00008 | 1.203 (1.073, 1.348) 0.00148 | |
| Q4 | 1.251 (1.155, 1.355) <0.00001 | 1.273 (1.165, 1.392) <0.00001 | 1.238 (1.100, 1.393) 0.00038 | |
| *P* for trend | <0.00001 | <0.00001 | 0.0009 | |
| Model 1: Non-adjusted  Model 2: Adjusted for age, gender, race, educational level, marital status, PIR  Model 3: Adjusted for age, gender, race, educational level, marital status, PIR, BMI, smoking status, alcohol consumption, hypertension, hyperlipidemia, diabetes, physical activity,anti-hypertensive drug usage,anti-hyperlipidemic drug usage and anti-diabetic drug usage. | | | |  |

**Table S6 Sensitivity Analysis of the Association Between FII and MASLD Risk After Exclusion of Participants with Diabetes**

| Exposure | Model 1 | Model 2 | Model 3 |
| --- | --- | --- | --- |
| FII Z-score | 1.145 (1.099, 1.192) <0.00001 | 1.209 (1.154, 1.268) <0.00001 | 1.125 (1.064, 1.190) 0.00003 |
| FII quartile |  |  |  |
| Q1 | 1 | 1 | 1 |
| Q2 | 1.188 (1.080, 1.308) 0.00043 | 1.207 (1.089, 1.337) 0.00033 | 1.216 (1.072, 1.380) 0.00234 |
| Q3 | 1.263 (1.147, 1.391) <0.00001 | 1.347 (1.213, 1.495) <0.00001 | 1.172 (1.030, 1.334) 0.01617 |
| Q4 | 1.354 (1.230, 1.490) <0.00001 | 1.555 (1.397, 1.729) <0.00001 | 1.370 (1.198, 1.567) <0.00001 |
| *P* for trend | <0.00001 | <0.00001 | 0.00003 |
| Model 1: Non-adjusted  Model 2: Adjusted for age, gender, race, educational level, marital status, PIR  Model 3: Adjusted for age, gender, race, educational level, marital status, PIR, BMI, smoking status, alcohol consumption, hypertension, hyperlipidemia, diabetes, physical activity,anti-hypertensive drug usage,anti-hyperlipidemic drug usage and anti-diabetic drug usage. | | | |

**Table S7** **Sensitivity Analysis of the Association Between FII and MASLD Risk After Exclusion of Participants with Hypertension**

| Exposure | Model 1 | Model 2 | Model 3 |
| --- | --- | --- | --- |
| FII Z-score | 1.153 (1.096, 1.212) <0.00001 | 1.206 (1.138, 1.278) <0.00001 | 1.110 (1.036, 1.189) 0.00288 |
| FII quartile |  |  |  |
| Q1 | 1 | 1 | 1 |
| Q2 | 1.210 (1.075, 1.362) 0.00164 | 1.213 (1.068, 1.377) 0.00297 | 1.249 (1.065, 1.466) 0.00639 |
| Q3 | 1.374 (1.221, 1.545) <0.00001 | 1.476 (1.298, 1.678) <0.00001 | 1.170 (0.992, 1.379) 0.06278 |
| Q4 | 1.386 (1.232, 1.559) <0.00001 | 1.593 (1.396, 1.817) <0.00001 | 1.361 (1.147, 1.615) 0.00040 |
| *P* for trend | <0.00001 | <0.00001 | 0.00191 |
| Model 1: Non-adjusted  Model 2: Adjusted for age, gender, race, educational level, marital status, PIR  Model 3: Adjusted for age, gender, race, educational level, marital status, PIR, BMI, smoking status, alcohol consumption, hypertension, hyperlipidemia, diabetes, physical activity,anti-hypertensive drug usage,anti-hyperlipidemic drug usage and anti-diabetic drug usage. | | | |

**Table S8 Sensitivity Analysis of the Association Between FII and MASLD Risk Using Complete Cases (Excluding Samples with Missing Covariates)**

| Exposure | Model 1 | Model 2 | Model 3 |
| --- | --- | --- | --- |
| FII Z-score | 1.152 (1.102, 1.205) <0.00001 | 1.197 (1.140, 1.257) <0.00001 | 1.082 (1.025, 1.141) 0.00402 |
| FII quartile |  |  |  |
| Q1 | 1 | 1 | 1 |
| Q2 | 1.141 (1.026, 1.270) 0.01475 | 1.160 (1.040, 1.293) 0.00760 | 1.163 (1.022, 1.323) 0.02217 |
| Q3 | 1.232 (1.109, 1.369) 0.00010 | 1.299 (1.165, 1.450) <0.00001 | 1.137 (0.997, 1.296) 0.05461 |
| Q4 | 1.460 (1.317, 1.619) <0.00001 | 1.699 (1.521, 1.898) <0.00001 | 1.369 (1.197, 1.566) <0.00001 |
| *P* for trend | <0.00001 | <0.00001 | 0.00002 |
| Model 1: Non-adjusted  Model 2: Adjusted for age, gender, race, educational level, marital status, PIR  Model 3: Adjusted for age, gender, race, educational level, marital status, PIR, BMI, smoking status, alcohol consumption, hypertension, hyperlipidemia, diabetes, physical activity,anti-hypertensive drug usage,anti-hyperlipidemic drug usage and anti-diabetic drug usage. | | | |

**Table S9 Sensitivity Analysis of the Association Between FII and MASLD Risk After Trimming the Top and Bottom 5% of FII Values**

| Exposure | Model 1 | Model 2 | Model 3 |
| --- | --- | --- | --- |
| FII Z-score | 1.144 (1.093, 1.198) <0.00001 | 1.182 (1.125, 1.242) <0.00001 | 1.083 (1.025, 1.144) 0.00452 |
| FII quartile |  |  |  |
| Q1 | 1 | 1 | 1 |
| Q2 | 1.202 (1.077, 1.341) 0.00099 | 1.223 (1.093, 1.368) 0.00043 | 1.204 (1.054, 1.375) 0.00612 |
| Q3 | 1.283 (1.151, 1.430) <0.00001 | 1.371 (1.225, 1.535) <0.00001 | 1.203 (1.052, 1.376) 0.00692 |
| Q4 | 1.457 (1.309, 1.622) <0.00001 | 1.654 (1.477, 1.853) <0.00001 | 1.369 (1.193, 1.570) <0.00001 |
| *P* for trend | <0.00001 | <0.00001 | 0.00003 |
| Model 1: Non-adjusted  Model 2: Adjusted for age, gender, race, educational level, marital status, PIR  Model 3: Adjusted for age, gender, race, educational level, marital status, PIR, BMI, smoking status, alcohol consumption, hypertension, hyperlipidemia, diabetes, physical activity,anti-hypertensive drug usage,anti-hyperlipidemic drug usage and anti-diabetic drug usage. | | | |

**Table S10 Sensitivity Analysis of the Association Between FII and MASLD Risk Using Multiple Imputation with Five Imputed Datasets and Pooled Results**

| Exposure | Model 1 | Model 2 | Model 3 |
| --- | --- | --- | --- |
| FII Z-score | 1.167 (1.127, 1.208) <0.00001 | 1.175 (1.132, 1.219) <0.00001 | 1.081 (1.037, 1.128) 0.00027 |
| FII quartile |  |  |  |
| Q1 | 1 | 1 | 1 |
| Q2 | 1.172 (1.080, 1.272) 0.00014 | 1.168 (1.075, 1.269) 0.00026 | 1.175 (1.063, 1.298) 0.00153 |
| Q3 | 1.341 (1.237, 1.454) <0.00001 | 1.350 (1.242, 1.467) <0.00001 | 1.195 (1.081, 1.321) 0.00052 |
| Q4 | 1.437 (1.326, 1.556) <0.00001 | 1.522 (1.397, 1.657) <0.00001 | 1.272 (1.146, 1.411) <0.00001 |
| *P* for trend | <0.00001 | <0.00001 | 0.00001 |
| Model 1: Non-adjusted  Model 2: Adjusted for age, gender, race, educational level, marital status, PIR  Model 3: Adjusted for age, gender, race, educational level, marital status, PIR, BMI, smoking status, alcohol consumption, hypertension, hyperlipidemia, diabetes, physical activity,anti-hypertensive drug usage,anti-hyperlipidemic drug usage and anti-diabetic drug usage. | | | |
